# Distribution of millipedes (Myriapoda, Diplopoda) along a forest interior – forest edge – grassland habitat complex

**DOI:** 10.3897/zookeys.510.8657

**Published:** 2015-06-30

**Authors:** Dávid Bogyó, Tibor Magura, Dávid D. Nagy, Béla Tóthmérész

**Affiliations:** 1 Department of Ecology, University of Debrecen, P.O. Box 71, Debrecen H-4010, Hungary; 2 Hortobágy National Park Directorate, P.O. Box 216, Debrecen H-4002, Hungary; 3 MTA-DE Biodiversity and Ecosystem Services Research Group, P.O. Box 71, Debrecen H-4010, Hungary

**Keywords:** Edge effect, soil arthropod, biodiversity, forest ecosystem

## Abstract

We studied the distribution of millipedes in a forest interior-forest edge-grassland habitat complex in the Hajdúság Landscape Protection Area (NE Hungary). The habitat types were as follows: (1) lowland oak forest, (2) forest edge with increased ground vegetation and shrub cover, and (3) mesophilous grassland. We collected millipedes by litter and soil sifting. There were overall 30 sifted litter and soil samples: 3 habitat types × 2 replicates × 5 soil and litter samples per habitats. We collected 9 millipede species; the most abundant species was *Glomeris tetrasticha*, which was the most abundant species in the forest edge as well. The most abundant species in the forest interior was *Kryphioiulus occultus*, while the most abundant species in the grassland was *Megaphyllum unilineatum*. Our result showed that the number of millipede species was significantly lower in the grassland than in the forest or in the edge, however there were no significant difference in the number of species between the forest interior and the forest edge. We found significantly the highest number of millipede individuals in the forest edge. There were differences in the composition of the millipede assemblages of the three habitats. The results of the DCCA showed that forest edge and forest interior habitats were clearly separated from the grassland habitats. The forest edge habitat was characterized by high air temperature, high soil moisture, high soil pH, high soil enzyme activity, high shrub cover and low canopy cover. The IndVal and the DCCA methods revealed the following character species of the forest edge habitats: *Glomeris tetrasticha* and *Leptoiulus cibdellus*. Changes in millipede abundance and composition were highly correlated with the vegetation structure.

## Introduction

Millipedes (Myriapoda, Diplopoda) are detrivores, feeding mainly on decaying plant material and they found usually under leaf litter (Hopkin and Read 1992). The typical and suitable habitats for millipedes are the deciduous temperate, subtropical and tropical forests where the population density of these animals can reach 1000 individuals per square metre (Golovatch and Kime 2009). Millipedes are also found in caves, deserts, grasslands, and in boreal (taiga) forests (Hopkin and Read 1992, Golovatch and Kime 2009). Changes in millipede’s assemblage composition, species diversity and population density correlate with vegetation structure (David et al. 1999). In habitat mosaics saprophagous macroarthropods have high species richness (David and Handa 2010). During recent destruction and fragmentation of forested habitats around the world, the percentage of forest edges rapidly grows (Saunders et al. 1991, David and Handa 2010). To monitor these processes, forest edges are in focus of the ecological and conservation researches (Ries et al. 2004, Tóthmérész et al. 2014).

Forest edges have distinctive structure with highly variable environmental conditions. Ries and Sisk (2004) defined edges as the boundary between patches with differing qualities. Edge effects create differences in biotic and abiotic factors on the borders of two habitats. At forest edges, changes in microclimate (radiation fluxes, wind, water flux) and decomposition processes can support changes in vegetation compared to the forest interior (Saunders et al. 1991, Gehlhausen et al. 2000, Riutta et al. 2012). A complex interaction of microclimate and biotic factors drives the changes in the vegetation across the forest edge to the forest interior (Gehlhausen et al. 2000). On the other hand, changes in the spatial distribution of animal species near habitat edges are also reported (Ries and Sisk 2004, Wirth et al. 2008). Classically, an increased species richness and density is predicted at habitat edges (Odum 1971, Magura et al. 2001, Wirth et al. 2008).

The edge effect on different arthropod taxa was frequently studied during the last decades (Coleoptera: Magura 2000, Lövei et al. 2006; Ewers and Didham 2008; Isopoda: Antonovic et al. 2012; multi-taxa approaches: Frainer and Duarte 2009, Biereinger et al. 2013). Millipedes were relatively little studied in the context of the edge effect, in spite of the rapid changes in temperature, soil structure and soil water content at forest edges (Camargo and Kapos 1995, Gehlhausen et al. 2000) and its major influence on litter decomposition processes in forests. But a few papers are available discussing the edge effect on millipedes (Gulička 1957, David et al. 1999, Tracz 2000, Weiermans and van Aarde 2003, Didham et al. 2009, Riutta et al. 2012).

The aim of our study was to test the edge effect hypothesis on millipedes, that predicts higher species diversity and population density in forest edges than in the adjacent habitats (Odum 1971, Magura et al 2001). Our study provided data on millipede abundance, diversity and assemblage composition in a habitat complex of forest interior, forest edge and grassland habitats. We also studied the relationships between saprophagous millipedes and selected environmental factors.

## Methods

We tested the edge effect hypothesis on millipedes in forest interior – forest edge – grassland habitat complex in Northeast Hungary. The study area is located in the Hajdúság Landscape Protection Area, about 3.5 km north-east from the centre of Vámospércs, 130–135 m above sea level (47°33'09"N, 21°56'18"E). The Hajdúság Landscape Protection Area is a mosaic of grasslands, forests and wetlands. Average annual temperature of this region is 9.6–9.8 °C, while average annual rainfall is 550–575 mm. Brown forest soils and sandy soils are the main soil types of the study area.

The studied habitat types were as follows: (1) a closed forest dominated by English oak (*Quercus robur*) and associated with narrow-leafed ash (Fraxinus angustifolia ssp. pannonica), field elm (*Ulmus minor*) and common alder (*Alnus glutinosa*) with shrubs and herbs and high percentage of canopy cover, (2) a forest edge with increased ground vegetation and dense shrub cover (dominated by *Crataegus monogyna*, *Prunus spinosa* and *Rhamnus catharticus*) as well as some invasive tree species, like *Robinia pseudoacacia*, and (3) a mesophilous grassland with dense herbaceous vegetation, dominated by *Festuca pratensis*, *Poa pratensis*, *Deschampsia caespitosa* and *Carex* species, together with Orchis laxiflora ssp. elegans and *Dactylorrhiza incarnata*.

This area was a forested area during the last decades. The mesophilous grassland is utilized for grazing (sheep and cattle). The age of trees in the forest interior and forest edge was 45–50 years, while the forest was unmanaged during the last 30 years. The density of trees was 250–300 trees/ha. The width of the forest edge was 6-14m. Non-native species occurred sparsely with single specimens in the forest edge.

We collected millipedes monthly (7 times from April to October in 2009; the year of 2009 was free from extreme weather conditions) during the vegetation period. Sampling of millipedes followed David et al. (1999) by litter and soil sampling using a metal frame (25 cm × 25 cm, and a depth of 5 cm). The material was sifted at the study site. The samples were sorted in the laboratory by hand within the next 24–48 hours. We collected samples along 100m long transects (parallel to the forest edge) per habitat type using 5 plots in each habitat. Along the 100 m long transects we signed 5 plot centers (25 m from each other), with 2.5 m radius around, from where every time a sample was taken at random. We collected 30 samples altogether in two spatial replicates, having a distance of 150m from each other (3 habitat types × 2 replicates × 5 samples). For identification we have used the works of Hauser and Voigtländer (2009) and Dziadosz (1968). Valid nomenclature was applied according to Enghoff (2013).

We selected 13 environmental variables to test the influence on millipede assemblages. We measured soil pH value, soil dehydrogenase activity, soil moisture, soil temperature (in a depth of 2 cm), air temperature and relative humidity on the surface in the study plots. For pH measurement soil solution was prepared from 6.0 g wet soil. Soil samples were put into plastic beakers and after it filled with 50 ml deionized water. The pH was measured with a digital measurement type Testo 206 (Testo AG, Germany). Soil dehydrogenase enzyme activity (indicator of microbiological activity through the oxidative metabolisms in soil) was determined using triphenyltetrazolium chloride method (Alef 1995).We measured soil moisture in the laboratory, comparing fresh and dried (at 105 °C for 24 hours) samples. Soil temperature, air temperature, and relative humidity were measured with field instrument Voltcraft DT-8820. We used the average of measurements (spring, summer, autumn). We also estimated the percentage cover of leaf litter, decaying wood materials, herbs, shrubs and canopy and measured the depth of the leaf litter within a circle of 250 cm radius around the plot centers. In addition we counted the number of woody plant species within a circle of 250 cm radius around the plot centers (Table 1).

**Table 1.** Average values of the environmental variables in the studied habitats.

	Grassland	Edge	Forest
Air temperature (°C)	26.60	24.20	21.61
Canopy cover (%)	0.00	50.30	69.80
Cover of decaying wood (%)	0.00	23.50	23.30
Cover of herbs (%)	93.80	25.30	23.50
Cover of leaf litter (%)	0.00	83.40	92.60
Dehydrogenase enzyme activity	0.37	0.31	0.29
Depth of leaf litter (cm)	0.00	2.47	2.88
Humidity (%)	69.88	66.08	71.30
Number of woody plant species	0.00	5.40	4.90
pH	8.54	7.83	7.40
Shrub cover (%)	0.00	66.10	34.80
Soil moisture (%)	33.28	28.17	16.97
Soil temperature (°C)	22.30	17.35	16.77

### Data analysis

Mixed Generalized Linear Model (GLMM) was used to test differences in the millipede abundance, species richness and Shannon diversity among the three habitat types (forest interior, forest edge, grassland). Factorial design was used; habitats and spatial replicates were regarded as factors. The response variables (millipede abundance, species richness, and Shannon diversity) were defined as a quasi-Poisson distribution with log link function (Zuur et al. 2009). When GLM revealed a significant difference between the means, a Tukey’s HSD test was performed for multiple comparisons among means.

The composition of the millipede assemblages along the forest interior – forest edge – grassland habitats was compared by hierarchical cluster analysis based on the abundance of millipedes using the Hellinger distance and the Ward fusion algorithm (Legendre and Legendre 1998). Quantitative character species of the studied habitats were identified using indicator value method (IndVal). This method quantifies the fidelity and specificity of the species in relation to groups of sites in a user-specified classification of sites, and tests for the statistical significance of the associations by permutation (Dufrene and Legendre 1997, Elek et al. 2001). Relationships between the studied environmental factors and the abundance of millipedes were examined using detrended canonical correspondence analysis (DCCA) by second order polynomials using the Canoco software package. Triplot scaling in the ordination was focused on the inter-species distances.

## Results

There were 999 specimens of millipedes (Diplopoda) identified to species level. In total 9 species of 4 families (Glomeridae, Julidae, Mastigophorophyllidae, Polydesmidae) were recorded from the study area (Table 2).

**Table 2.** List of millipede species with their abundances recorded in the studied habitats.

	Grassland	Edge	Forest
*Brachyiulus bagnalli* (Broelemann, 1924)		3	5
*Brachydesmus superus* Latzel, 1884		1	10
*Glomeris tetrasticha* Brandt, 1833	1	382	33
*Julus terrestris* Linnaeus, 1758	6	8	8
*Kryphioiulus occultus* (C.L. Koch, 1847)	7	121	141
*Leptoiulus cibdellus* (Chamberlin, 1921)	1	34	2
*Mastigona bosniensis* (Verhoeff, 1897)		104	38
*Megaphyllum unilineatum* (C.L. Koch, 1838)	16	21	12
*Polydesmus complanatus* (Linnaeus, 1761)		22	23
Total	31	696	272

The forest edge and forest interior habitats were more species rich (9 species) than the grassland. 5 species of millipedes was found in the grassland habitat. *Glomeris tetrasticha*, *Julus terrestris*, *Kryphioiulus occultus*, *Leptoiulus cibdellus* and *Megaphyllum unilineatum* were recorded in all studied habitats. The following millipede species were the most frequent: *Glomeris tetrasticha* (416 individuals), *Kryphioiulus occultus* (269 individuals) and *Mastigona bosniensis* (142 individuals), while *Brachyiulus bagnalli* (8 individuals) and *Brachydesmus superus* (11 individuals) had the lowest total abundance in the studied habitats. The most abundant millipede species, *Glomeris tetrasticha* represented 41.6% of the total millipede catch. The same species was also the most frequent millipede (54.9% of the millipede individuals) in the edge habitat. The highest total abundance of millipedes was found in the edge habitat (696 individuals), while the lowest number of millipede individuals (31) was found in the grassland habitat.

**Figure 1.**
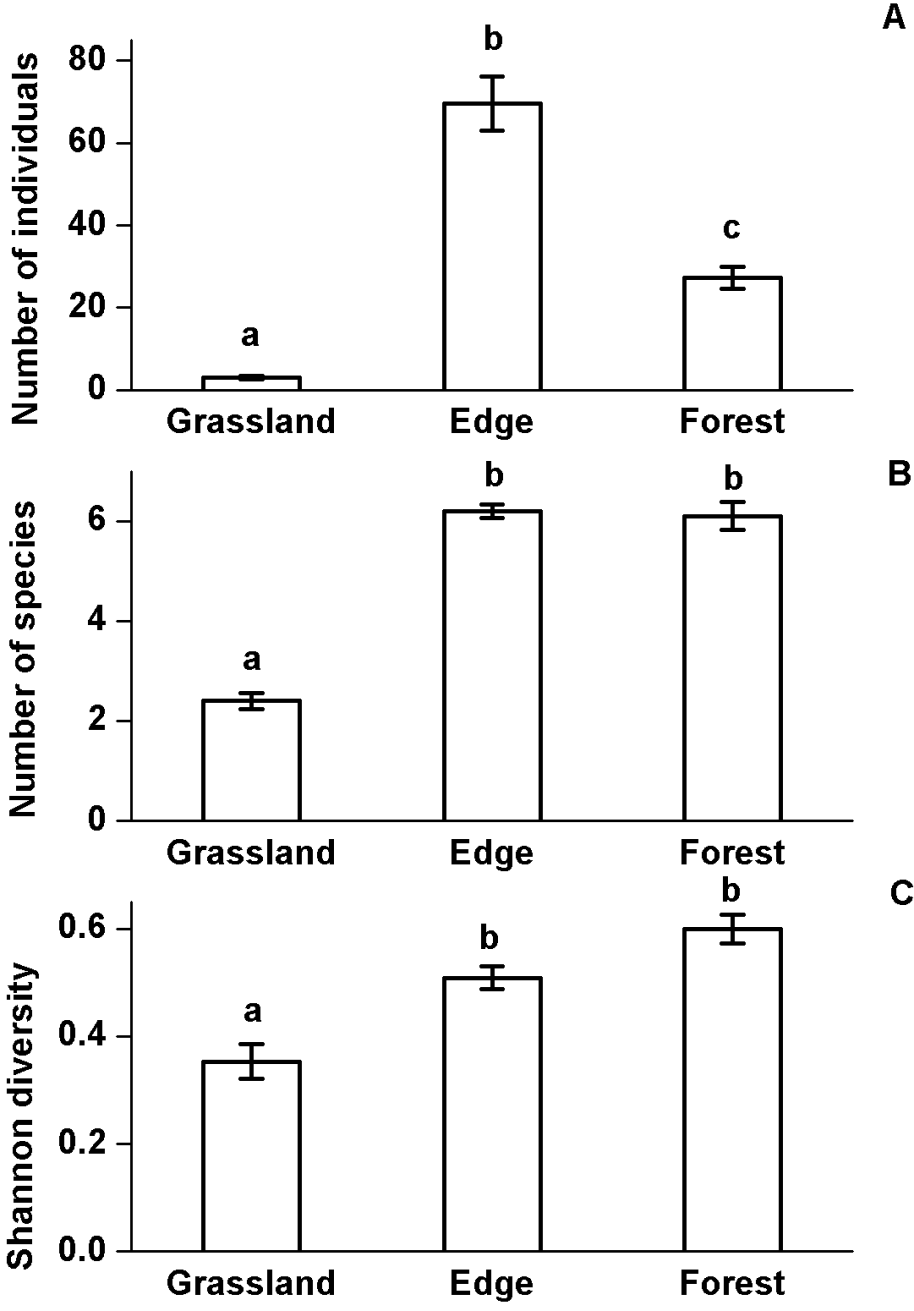
Millipede abundance, species richness and Shannon diversity at the studied habitats. Mean values (±SD) of the overall millipede abundance (**A**), species richness (**B**) and Shannon diversity (**C**) per samples at the studied habitats. Different letters indicate signiﬁcant differences by Tukey test.

The number of millipede individuals was significantly higher in the forest edge than in the grassland and forest interior. Furthermore, the number of millipede individuals was significantly higher in the forest interior than in the grassland (Chi^2^=179.275; df=2; p<0.0001; Figure 1a). The number of millipede species was significantly lower in the grassland than in the forest edge and forest interior (Chi^2^=231.974; df=2; p<0.0001; Figure 1b), while there was no difference between the number of millipede species in the forest edge and forest interior. A similar result was found for the Shannon diversity of the millipede assemblage which was significantly lower in the grassland habitat than in the forest edge and forest interior (Chi^2^=40.849; df=2; p<0.0001; Figure 1c). There was no significant difference between the diversity of millipede assemblage in the forest edge and forest interior habitats.

**Figure 2.**
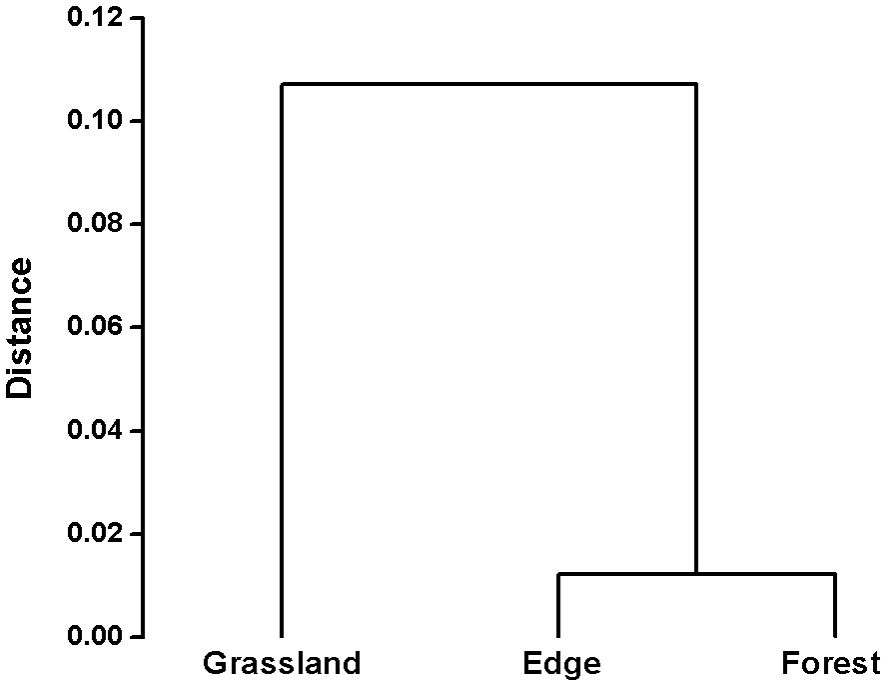
Hierarchical cluster analysis of millipede assemblages of the studied habitats using Hellinger distance and Ward fusion method.

The millipede assemblages of the studied habitats formed two separated groups by hierarchical cluster analysis (Figure 2). The first group included the grassland plots, while the second one included the forest edge’s and forest interior’s plots.

**Figure 3.**
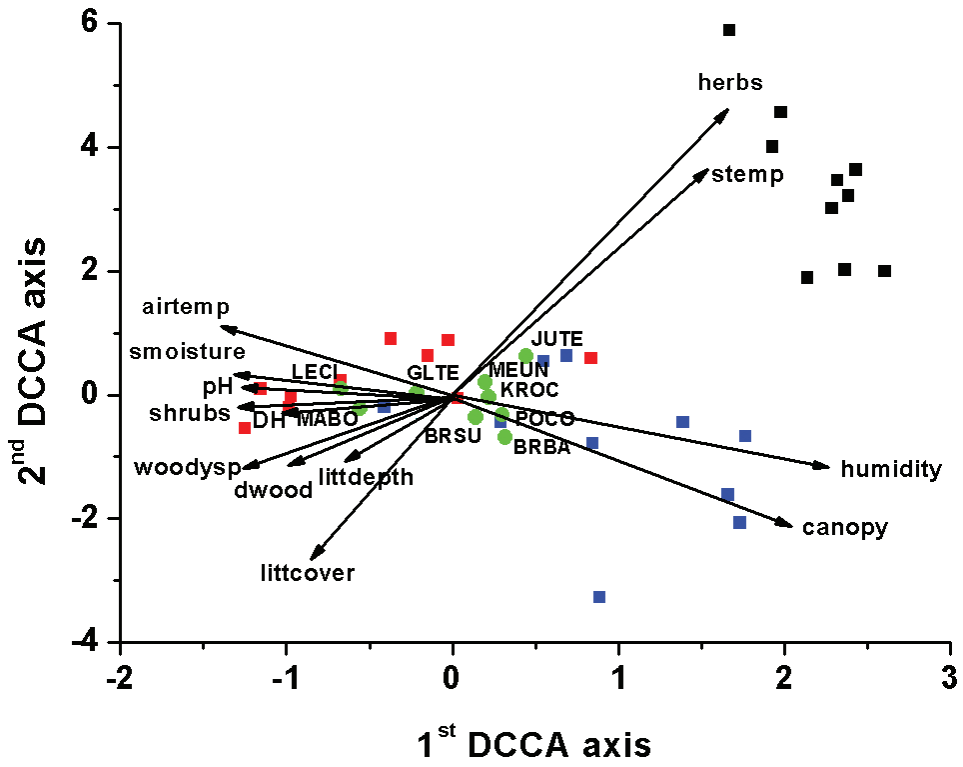
DCCA analysis for the millipede species of the study area. Squares represent the sampled habitats (blue squares: samples from the forest interior habitat; red squares: samples from the forest edge habitat; black squares: samples from the grassland habitat).The arrows denote the increase of the value of the environmental variables (airtemp: air temperature on the surface; canopy: canopy cover; DH: soil dehydrogenase enzyme activity; dwood: cover of decaying wood material; herbs: cover of herbs; littcover: cover of leaf litter; littdepth: depth of leaf litter; humidity: relative humidity on the surface; pH: soil pH; shrubs: cover of shrubs; stemp: soil temperature at 2cm depth; smoisture: soil moisture; woodysp: number of woody plant species). Green circles and the four-letter abbreviations indicate the millipede species (BRBA: *Brachyiulus bagnalli*; BRSU: *Brachydesmus superus*; GLTE: *Glomeris tetrasticha*; JUTE: *Julus terrestris*; KROC: *Kryphioiulus occultus*; LECI: *Leptoiulus cibdellus*; MABO: *Mastigona bosniensis*; MEUN: *Megaphyllum unilineatum*; POCO: *Polydesmus complanatus*).

We identified two groups of quantitative character species by the IndVal method for the studied habitats (Table 3): (1) species characteristic of the forest edge habitat (*Leptoiulus cibdellus*); (2) species characteristic of the forest edge and forest interior habitats (*Glomeris tetrasticha*, *Kryphioiulus occultus*, *Mastigona bosniensis*, *Polydesmus complanatus* Linnaeus, 1761). The result of the DCCA showed a marked separation of the three studied habitats (Figure 3). The forest interior habitat was characterized by high canopy cover, high relative humidity and low air temperature, while *Brachydesmus superus* was associated with this habitat. The forest edge habitat was characterized by high air temperature, high soil moisture, high soil pH, high soil dehydrogenase activity, high shrub cover and low canopy cover. According to the DCCA results, *Glomeris tetrasticha* and *Leptoiulus cibdellus* were associated with the forest edge. The grassland habitat differed from the forest edge and forest interior habitats, which were more similar to each other. The grassland habitat plots were located on the right upper region, whereas the forest edge plots on the left center region, and the forest interior plots on the right lower part of the ordination plot. The grassland habitat was characterized by high soil temperature and high cover of herbs as well as the absence of the leaf litter and dead wood. Similarly to the results of the IndVal method, no species was associated with this habitat. *Megaphyllum unilineatum* and *Julus terrestris* were situated near the origin, indicating no clear preference for any of the studied habitats. The remaining four species (*Mastigona bosniensis*, *Kryphioiulus occultus*, *Polydesmus complanatus*, *Brachyiulus bagnalli*), similarly to the IndVal results, associated with the forest edge and forest interior habitats.

**Table 3.** Habitat preference and quantitative character values of the millipede species presented with more than 30 individuals altogether. The IndVal column shows the species character value for the corresponding cluster level. Notations: * - p<0.05. A: the number of specimens present, B: the number of samples where the species is present in the sample group.

Species	IndVal	p	Grassland		Edge		Forest	
			A	B	A	B	A	B
Forest edge								
*Leptoiulus cibdellus*	82.7	*	1	1	34	9	2	2
Forest edge and forest interior								
*Glomeris tetrasticha*	99.5	*	1	1	382	10	33	10
*Mastigona bosniensis*	95	*	0	0	104	10	38	9
*Kryphioiulus occultus*	90.2	*	7	7	121	9	141	10
*Polydesmus complanatus*	85	*	0	0	22	8	23	9

## Discussion

We found a relatively low number of millipede species in the complex of three habitat types in a protected area of Hungary, which represents approximately 9% of the Hungarian millipede fauna (Bogyó et al. 2012). The majority of the millipede species were Central-European species preferring forested habitats (Korsós 1994, Voigtländer 2011, Bogyó et al. 2012, Enghoff 2013), which confirmed the relatively undisturbed conditions in the Hajdúság Landscape Protection Area. The most abundant species in the study and in the forest edge habitat was *Glomeris tetrasticha*. This is a widespread montane species in eastern part of Central Europe, preferring humid conditions, while the occurrences in Hungary were formerly considered as glacial relicts (Korsós 1994). However it is not uncommon that the species occurs in different lowland forests of Northeastern Hungary (Szlávecz and Loksa 1991, Bogyó et al. 2012). The dominance of glomerid species in forested habitats was pointed out by other authors in contrast with the dominance of julid species in grasslands (Tajovský 1990, David et al. 1999). The second and third most abundant species (*Kryphioiulus occultus* and *Mastigona bosniensis*) are found in a wide range of wooded and more or less opened habitats across Central-Europe (Korsós 1994, Voigtländer 2011, Bogyó et al. 2012).

The aim of the study was to reveal a relationship between millipedes (abundance, species richness, diversity and assemblage composition) and the vegetation structure along a forest interior-forest edge-grassland gradient. We found that the total millipede abundance was highest in the forest edge which supports the classical edge effect hypothesis (Odum 1971, Magura et al. 2001, Wirth et al. 2008). Such pattern was expected, but distribution of some species is slightly different from the pattern described in the literature. Our results are also coherent with the predictions of Ries and Sisk (2004). In the forest edge habitat millipedes can benefit from higher temperatures and higher openness compared to the forest interior, but they also benefit from higher percentage of leaf litter cover and thicker leaf litter layer, as well as the higher amount of dead wood and soil moisture than in the adjacent habitats. A review on edge effect (Wirth et al. 2008) reported that the majority of the studies showed a positive edge effect on the abundance of herbivorous arthropods. This work highlighted that edges can have a positive effect on the palatability of resources by affecting fluxes of nutrients, while on the other hand edges represent high-resource environments for forest plants (according to higher light and nutrient availability) which leads to an increase of leaf productivity. However, millipedes are not herbivores, but saprophages, and they are strongly related to the quality and quantity of decaying plant material (Hopkin and Read 1992, Stašiov et al. 2012). Previous studies of edge effect on millipede abundance also reported a positive effect. In a Mediterranean landscape of France, David et al. (1999) found higher millipede and woodlouse population densities and biomass in semi-opened sites (covered by shrubs within the height of 0.25m and 2m) than in forested areas (oak forests with more than 2m high trees). Similarly, high millipede abundance was reported from ecotones of Poland (Tracz 2000). Tracz (2000) studied three sites, where the core forest was a beech-oak mixed forest (*Fago-Quercetum petreae*) and the ecotone was represented by high dominance of the (1) common broom (*Cytisus scopariius*) or (2) common aspen (*Populus tremula*) or (3) beech (*Fagus silvatica*). In the edge habitats of tawa (*Beilschmiedia tawa*) forests of New Zeeland a positive edge effect on millipede abundance was also demonstrated. In contrary, other studies showed no difference in millipede abundance between forest and forest edge habitats in Brasil (Frainer and Duarte 2009), in South-Africa (Weiermans and van Aarde 2003) and in the United Kingdom (Riutta et al. 2012).

Our study showed no significant edge effect on the species richness and diversity of millipedes. Millipede species richness and diversity was higher in the forest interior and forest edge than in the grassland, but there was no difference between the two forested habitats (forest interior, and forest edge). A positive edge effect on species richness and/or diversity in forest edges was reported in previous studies on arthropod taxa (Magura et al. 2000, 2001, Magura 2002, Wirth et al. 2008, Antonovic et al. 2012). However, other studies showed an opposite trend or reported no significant edge effect on species richness and/or diversity of arthropods (Dangerfield et al. 2003, Ries et al. 2004, Wirth et al. 2008). In case of millipedes, David et al. (1999) found significantly higher species diversity in semi-open sites compared with forested and open habitats, which was interpreted as an edge effect. High diversity of millipedes was reported from ecotonal zones of forests (Tracz 2000) and plant borders of city gardens (Smith et al. 2006). On the other hand, Riutta et al. (2012) showed significantly higher millipede species richness in the forest interior than in the forest edge in a temperate mixed deciduous forest. No clear trend was found in millipede species richness of coastal dune forests and its edge habitats in South-Africa (Weyermans and van Aarde 2003). Former studies (David and Handa 2010) predicted higher millipede diversity in habitat mosaics with higher heterogeneity, because of the presence of different habitat patches. In our study, we do not detected higher millipede diversity in the forest edge habitat compared to the forest interior.

The millipede assemblages of the studied habitats clearly separated from each other, based on the abundance of the millipede species. The millipede assemblages formed two main clusters: the first included the grassland habitat, the second included the two forested habitats. However, forested habitats (forest edge and forest interior) also showed a clear separation from each other. It is known, that saprophagous macroarthropod assemblages are changing with the change of vegetation structure on a landscape scale, or on a smaller scale (David et al. 1999, Wytwer et al. 2009, David and Handa 2010, Foster and Claeson 2011). Moreover, even the tree species composition can significantly affect millipede assemblage composition (Stašiov et al. 2012). The influence of the habitat type on millipede assemblages can override the effect of the successional stage also (Schreiner et al. 2012).

Using the IndVal method we found significant character species for the studied habitats. The grassland habitat had no significant character species. The forest edge was characterized by *Leptoiulus cibdellus*, a species preferring humid, woodland habitats with a Northern and Central-East European distribution (Korsós 1994, Bogyó et al. 2012, Enghoff 2013). Species associated with the forest edge and forest interior habitats (*Glomeris tetrasticha*, *Kryphioiulus occultus*, *Mastigona bosniensis*, *Polydesmus complanatus*) are more or less natural woodland species. According to the literature (Korsós 1994, Voigtländer 2011, Bogyó et al. 2012, Enghoff 2013), some of these species are described from opened and xeric woodland habitats (*Kryphioiulus occultus*, *Mastigona bosniensis*), while others prefer more closed and humid forests (*Glomeris tetrasticha*, *Polydesmus complanatus*). Other works in Europe also listed characteristic species of edge habitats (David et al. 1999, Tracz 2000). David et al. (1999) found that semi-open sites were dominated by a *Glomeris* species, while the dominance of the family Julidae was pointed out by Tracz (2000), supporting the hypothesis that juloid morphotype is the best adaptation to various adverse environments. In our study, the most abundant millipede species in the edge habitat was *Glomeris tetrasticha*. In the forest edge the abundance of this species was twice as much than the total abundance of millipedes of the Julidae family.

The results of the DCCA showed that forest edge and forest interior habitats are clearly separated from the grassland habitats. Forest edge and forest interior offer more suitable habitat for millipedes with high amount of leaf litter (both cover and depth of leaf litter), dead wood, canopy cover, as well as with more humid microclimate. The studied forest edge habitat was characterized by high air temperature, high soil moisture, high soil pH and low canopy cover (higher openness) which are key factors affecting millipede assemblages in forested habitats (Hopkin and Read 1992, David 1999, Stašiov 2005, 2009). Lower abundance of saprophagous macroarthropods in forested sites and decreasing abundance by increasing oak cover was observed by David et al. (1999) in France. In our study the edge habitat was also characterized by high percentage of shrub cover as well as high number of woody plant species. Even if the edge habitat has lower percentage of canopy cover and higher air temperature, the saplings (as well as the shrubs) can protect the forest edge from desiccation, leading to high soil moisture (Camargo and Kapos 1995). Lindsay and French (2006) reported that the natural shrublands infested with a dense population of the non-native bitou bush in Australia resulted in an increase in millipede abundance, possibly as a result of a changing microclimate with moister environment. The moister microhabitats could be favorable for millipede species preferring humid conditions, like the two characteristic species of the forest edge, *Glomeris tetraticha* and *Leptoiulus cibdellus*. In our study, the changes in canopy and shrub cover (as well as light conditions) combined with high soil moisture and diverse leaf litter could lead to the significantly higher millipede abundance in the forest edge compared to the adjacent habitats. High values of dehydrogenase activity were also found in the forest edge which is usually has a positive correlation with millipede abundance (Tripathi et al. 2013, Vasconcellos et al. 2013).

## Conclusion

The results of the study revealed that millipede assemblages altered in a short distance along the grassland-forest edge-forest habitat complex. Our study showed a positive edge effect on millipede abundance and assemblage composition in the studied natural habitats. We found significant edge-associated millipede species by IndVal method. On the other hand there was no edge effect on millipede species richness and diversity. Our results support the former findings that some millipede species may be specialized to natural forest edge habitats. Forest edges may have key habitats in the conservation of millipedes during the next decades.
